# 1-Dodecyl-1*H*-benzo[*d*]imidazol-2(3*H*)-one

**DOI:** 10.1107/S1600536812041189

**Published:** 2012-10-06

**Authors:** Dounia Belaziz, Youssef Kandri Rodi, Fouad Ouazzani Chahdi, El Mokhtar Essassi, Mohamed Saadi, Lahcen El Ammari

**Affiliations:** a Laboratoire de Chimie Organique Appliquée, Université Sidi Mohamed, Ben Abdallah, Faculté des Sciences et Techniques, Route d’immouzzer, BP 2202 Fès, Morocco; b Laboratoire de Chimie Organique Hétérocyclique URAC21, Faculté des Sciences, Université Mohammed V-Agdal, Avenue Ibn Battouta, BP 1014, Rabat, Morocco; c Institute of Nanmaterials and Nanotechnology, MASCIR, Rabat, Morocco; d Laboratoire de Chimie du Solide Appliquée, Faculté des Sciences, Université Mohammed V-Agdal, Avenue Ibn Battouta, BP 1014, Rabat, Morocco

## Abstract

In the title compound, C_19_H_30_N_2_O, the fused ring system is essentially planar, the maximum deviation from the mean plane being 0.013 (2) Å for the N atom bearing the dodecyl chain. The 1-dodecyl group is almost perpendicular to the 1*H*-benzo[*d*]imidazol-2(3*H*)-one plane as indicated by the dihedral angle of 82.9 (2)°between planes through the fused ring system and the first three C atoms of the chain. The C—C—C—C torsion angles (about ±179°) of the dodecyl group indicate an anti­periplanar conformation. In the crystal, inversion dimers are formed by pairs of N—H⋯O hydrogen bonds.

## Related literature

For pharmacological and biochemical properties of benzimidazoles and their derivatives, see: Al Muhaimeed (1997 ▶); Scott *et al.* (2002 ▶); Nakano *et al.* (2000 ▶); Zhu *et al.* (2000 ▶); Zarrinmayeh *et al.* (1998 ▶). For compounds with closely related structures, see: Ouzidan *et al.* (2011 ▶); Kandri Rodi *et al.* (2011 ▶); Belaziz *et al.* (2012 ▶).
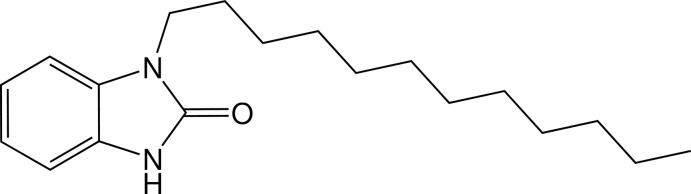


## Experimental

### 

#### Crystal data

C_19_H_30_N_2_O
*M* *_r_* = 302.45
Monoclinic, 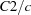

*a* = 38.3223 (14) Å
*b* = 4.8318 (2) Å
*c* = 21.9831 (8) Å
β = 117.843 (2)°
*V* = 3599.3 (2) Å^3^
*Z* = 8
Mo *K*α radiation
μ = 0.07 mm^−1^
*T* = 296 K
0.47 × 0.31 × 0.14 mm

#### Data collection

Bruker X8 APEX Diffractometer
29002 measured reflections
4637 independent reflections
3179 reflections with *I* > 2σ(*I*)
*R*_int_ = 0.028

#### Refinement

*R*[*F* ^2^ > 2σ(*F* ^2^)] = 0.045
*wR*(*F* ^2^) = 0.141
*S* = 1.01
4637 reflections
199 parameters
H-atom parameters constrained
Δρ_max_ = 0.21 e Å^−3^
Δρ_min_ = −0.21 e Å^−3^

### 

Data collection: *APEX2* (Bruker, 2005 ▶); cell refinement: *SAINT* (Bruker, 2005 ▶); data reduction: *SAINT*; program(s) used to solve structure: *SHELXS97* (Sheldrick, 2008 ▶); program(s) used to refine structure: *SHELXL97* (Sheldrick, 2008 ▶); molecular graphics: *ORTEP-3 for Windows* (Farrugia, 1997 ▶); software used to prepare material for publication: *PLATON* (Spek, 2009 ▶) and *publCIF* (Westrip, 2010 ▶).

## Supplementary Material

Crystal structure: contains datablock(s) I. DOI: 10.1107/S1600536812041189/im2402sup1.cif

Structure factors: contains datablock(s) I. DOI: 10.1107/S1600536812041189/im2402Isup2.hkl

Supplementary material file. DOI: 10.1107/S1600536812041189/im2402Isup3.cml

Additional supplementary materials:  crystallographic information; 3D view; checkCIF report

## Figures and Tables

**Table 1:** Hydrogen-bond geometry (Å, °)

*D*—H⋯*A*	*D*—H	H⋯*A*	*D*⋯*A*	*D*—H⋯*A*
N1—H1⋯O1^i^	0.86	1.97	2.815 (1)	168

Symmetry code: (i) 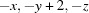.

**Fig. 1.**
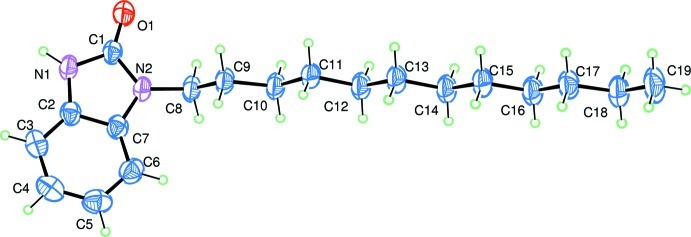
Molecular structure of the title compound with displacement ellipsoids drawn at the 50% probability level. H atoms are represented as small circles.

**Fig. 2.**
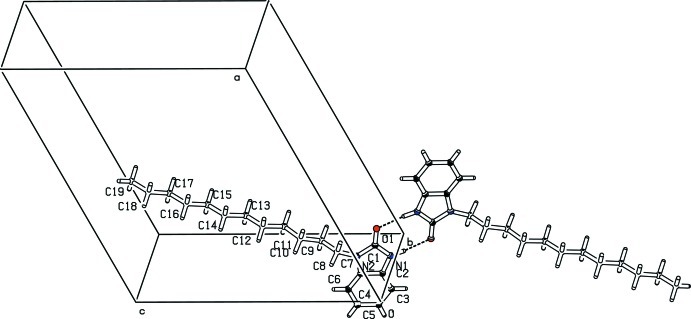
Inversion dimer with molecules linked by N—H···O hydrogen bonds.

## supplementary crystallographic information

### Comment

Benzimidazoles and their derivatives exhibit a number of important pharmacological properties, such as antihistaminic (Al Muhaimeed, 1997) anti-ulcerative (Scott *et al.*, 2002) and antiallergic (Nakano *et al.*, 2000). In addition, benzimidazole derivatives are effective against the human cytomegalovirus (HCMV) (Zhu *et al.*, 2000) and are also efficient selective neuropeptide Y Y1 receptor antagonists (Zarrinmayeh *et al.*, 1998).

In a previous study, we reacted benzimidazol-2-one with octyl bromide in the presence of a catalytic quantity of tetra-n-butylammonium bromide under mild conditions to form 1-octyl-1*H*-benzo[*d*]imidazol-2(3*H*)-one (Belaziz *et al.*, 2012). The study has been extended to the synthesis of a new benzimidazol-2-one derivative by action of dodecyl bromide with 1*H*-benzo[*d*]imidazol-2(3*H*)-one to form the title compound (Scheme 1).

The molecular structure of 1-dodecyl-1*H*-benzo[*d*]imidazol-2(3*H*)-one is built up from fused six-and five-membered rings linked to a C_12_H_25_ chain as shown in Fig. 1. The fused-ring system is essentially planar, with a maximum deviation of -0.013 (2) Å for N2. The dodecyl group is almost perpendicular to the 1*H*-benzo[*d*]imidazol-2(3*H*)-one plane as indicated by the dihedral angle between planes (C8 C9 C10) and (N1 N2 C1 to C7) of 82.9 (2)° and by the torsion angle (C7 N2 C8 C9) = -84.3 (2)°. In the crystal structure, inversion dimers are formed by N—H···O hydrogen bonds. in the way to form dimers (Fig. 2).

The structure of the title compound is almost identical to that observed for the following molecules: 1-nonyl-1*H*-benzimidazol-2(3*H*)-one, 1-octyl-1*H*-benzimidazol-2(3*H*)-one and 5-chloro-1-nonyl-1*H*-benzimidazol-2(3*H*)-one (Ouzidan *et al.*, 2011, Kandri Rodi *et al.* 2011). Nevertheless, the different lengths of the chains leads to different unit cells with different crystal symmetry.

### Experimental

To 1*H*-benzo[*d*]imidazol-2(3*H*)-one (0.2 g, 1.49 mmol), potassium carbonate (0.41 g, 2.98 mmol) and tetra-n-butylammonium bromide (0.05 g, 0.15 mmol) in DMF (15 ml) was added dodecyl bromide (0.30 ml, 1.78 mmol). Stirring was continued at room temperature for 6 h. The salt was removed by filtration and the filtrate concentrated under reduced pressure. The residue was separated by chromatography on a column of silica gel with ethyl acetate/hexane (1/2) as eluent (yield: 65%). The compound was recrystallized from hexan/acetate to give colourless crystals.

### Refinement

H atoms were located in a difference map and treated as riding with N—H = 0.86 Å, C—H = 0.93 Å (aromatic), C—H = 0.97 Å (methylene) and C—H = 0.96 Å (methyl) with *U*_iso_(H) = 1.2 *U*_eq_ (N—H, aromatic, methylene) and *U*_iso_(H) = 1.5 *U*_eq_(methyl).

### Crystal data

**Table:**

C_19_H_30_N_2_O	*F*(000) = 1328
*M**_r_* = 302.45	*D*_x_ = 1.116 Mg m^−^^3^
Monoclinic, *C*2/*c*	Melting point: 346.5 K
Hall symbol: -C 2yc	Mo *K*α radiation, λ = 0.71073 Å
*a* = 38.3223 (14) Å	Cell parameters from 4637 reflections
*b* = 4.8318 (2) Å	θ = 2.4–28.7°
*c* = 21.9831 (8) Å	µ = 0.07 mm^−^^1^
β = 117.843 (2)°	*T* = 296 K
*V* = 3599.3 (2) Å^3^	Needle, colourless
*Z* = 8	0.47 × 0.31 × 0.14 mm

### Data collection

**Table:**

Bruker X8 APEX Diffractometer	3179 reflections with *I* > 2σ(*I*)
Radiation source: fine-focus sealed tube	*R*_int_ = 0.028
Graphite monochromator	θ_max_ = 28.7°, θ_min_ = 2.4°
φ and ω scans	*h* = −51→51
29002 measured reflections	*k* = −6→6
4637 independent reflections	*l* = −29→28

### Refinement

**Table:**

Refinement on *F*^2^	Primary atom site location: structure-invariant direct methods
Least-squares matrix: full	Secondary atom site location: difference Fourier map
*R*[*F*^2^ > 2σ(*F*^2^)] = 0.045	Hydrogen site location: difference Fourier map
*wR*(*F*^2^) = 0.141	H-atom parameters constrained
*S* = 1.01	*w* = 1/[σ^2^(*F*_o_^2^) + (0.0721*P*)^2^ + 1.030*P*] where *P* = (*F*_o_^2^ + 2*F*_c_^2^)/3
4637 reflections	(Δ/σ)_max_ < 0.001
199 parameters	Δρ_max_ = 0.21 e Å^−^^3^
0 restraints	Δρ_min_ = −0.21 e Å^−^^3^

### Special details

**Table:**

Geometry. All s.u.'s (except the s.u. in the dihedral angle between two l.s. planes) are estimated using the full covariance matrix. The cell s.u.'s are taken into account individually in the estimation of s.u.'s in distances, angles and torsion angles; correlations between s.u.'s in cell parameters are only used when they are defined by crystal symmetry. An approximate (isotropic) treatment of cell s.u.'s is used for estimating s.u.'s involving l.s. planes.
Refinement. Refinement of *F*^2^ against all reflections. The weighted *R*-factor *wR* and goodness of fit *S* are based on *F*^2^, conventional *R*-factors *R* are based on *F*, with *F* set to zero for negative *F*^2^. The threshold expression of *F*^2^ > 2σ(*F*^2^) is used only for calculating *R*-factors(gt) *etc*. and is not relevant to the choice of reflections for refinement. *R*-factors based on *F*^2^ are statistically about twice as large as those based on *F*, and *R*- factors based on all data will be even larger.

### Fractional atomic coordinates and isotropic or equivalent isotropic displacement parameters (Å^2^)

**Table:**

	*x*	*y*	*z*	*U*_iso_*/*U*_eq_	
N2	0.03233 (3)	0.5579 (2)	0.13178 (5)	0.0394 (2)	
N1	−0.01601 (3)	0.7314 (2)	0.03782 (5)	0.0398 (2)	
H1	−0.0289	0.8297	0.0014	0.048*	
O1	0.04750 (2)	0.91076 (19)	0.07646 (4)	0.0472 (2)	
C11	0.13888 (4)	0.6345 (3)	0.37748 (6)	0.0440 (3)	
H11A	0.1187	0.6113	0.3920	0.053*	
H11B	0.1428	0.8315	0.3747	0.053*	
C7	−0.00182 (3)	0.4187 (2)	0.12105 (6)	0.0386 (3)	
C9	0.08398 (3)	0.6105 (3)	0.25353 (6)	0.0431 (3)	
H9A	0.0644	0.5596	0.2679	0.052*	
H9B	0.0844	0.8108	0.2510	0.052*	
C15	0.24328 (4)	0.5988 (3)	0.62951 (6)	0.0514 (3)	
H15A	0.2457	0.7986	0.6294	0.062*	
H15B	0.2233	0.5564	0.6435	0.062*	
C12	0.17715 (4)	0.5077 (3)	0.43133 (6)	0.0484 (3)	
H12A	0.1737	0.3089	0.4317	0.058*	
H12B	0.1977	0.5418	0.4182	0.058*	
C1	0.02366 (3)	0.7522 (2)	0.08097 (5)	0.0371 (3)	
C13	0.19082 (4)	0.6180 (3)	0.50358 (6)	0.0498 (3)	
H13A	0.1705	0.5816	0.5171	0.060*	
H13B	0.1940	0.8170	0.5032	0.060*	
C8	0.07235 (3)	0.4891 (3)	0.18297 (6)	0.0424 (3)	
H8A	0.0906	0.5557	0.1671	0.051*	
H8B	0.0749	0.2894	0.1870	0.051*	
C2	−0.03246 (3)	0.5297 (2)	0.06114 (6)	0.0383 (3)	
C10	0.12435 (4)	0.5075 (3)	0.30657 (6)	0.0463 (3)	
H10A	0.1232	0.3081	0.3104	0.056*	
H10B	0.1433	0.5476	0.2902	0.056*	
C17	0.29647 (4)	0.5852 (3)	0.75462 (6)	0.0513 (3)	
H17A	0.2983	0.7854	0.7541	0.062*	
H17B	0.2769	0.5392	0.7693	0.062*	
C14	0.22938 (4)	0.4922 (3)	0.55680 (6)	0.0517 (3)	
H14A	0.2497	0.5298	0.5433	0.062*	
H14B	0.2262	0.2930	0.5567	0.062*	
C16	0.28233 (4)	0.4785 (3)	0.68205 (6)	0.0522 (3)	
H16A	0.2798	0.2788	0.6823	0.063*	
H16B	0.3022	0.5199	0.6678	0.063*	
C6	−0.00851 (4)	0.2120 (3)	0.15747 (7)	0.0493 (3)	
H6	0.0118	0.1406	0.1977	0.059*	
C3	−0.07051 (4)	0.4301 (3)	0.03570 (7)	0.0490 (3)	
H3	−0.0910	0.5015	−0.0044	0.059*	
C5	−0.04689 (4)	0.1145 (3)	0.13174 (7)	0.0553 (4)	
H5	−0.0524	−0.0248	0.1552	0.066*	
C18	0.33590 (4)	0.4716 (3)	0.80666 (7)	0.0616 (4)	
H18A	0.3555	0.5176	0.7921	0.074*	
H18B	0.3341	0.2714	0.8072	0.074*	
C19	0.34981 (5)	0.5790 (4)	0.87888 (7)	0.0774 (5)	
H19A	0.3750	0.4990	0.9090	0.116*	
H19B	0.3310	0.5294	0.8944	0.116*	
H19C	0.3522	0.7768	0.8791	0.116*	
C4	−0.07710 (4)	0.2207 (3)	0.07186 (8)	0.0554 (4)	
H4	−0.1024	0.1494	0.0556	0.066*	

### Atomic displacement parameters (Å^2^)

**Table:**

	*U*^11^	*U*^22^	*U*^33^	*U*^12^	*U*^13^	*U*^23^
N2	0.0357 (5)	0.0440 (5)	0.0272 (4)	0.0014 (4)	0.0053 (4)	0.0030 (4)
N1	0.0355 (5)	0.0458 (6)	0.0282 (4)	0.0032 (4)	0.0065 (4)	0.0038 (4)
O1	0.0404 (4)	0.0546 (5)	0.0378 (4)	−0.0026 (4)	0.0110 (4)	0.0066 (4)
C11	0.0434 (6)	0.0463 (7)	0.0299 (5)	−0.0011 (5)	0.0067 (5)	0.0014 (5)
C7	0.0416 (6)	0.0397 (6)	0.0304 (5)	0.0009 (5)	0.0134 (5)	−0.0038 (4)
C9	0.0410 (6)	0.0450 (7)	0.0305 (6)	0.0040 (5)	0.0061 (5)	0.0011 (5)
C15	0.0462 (7)	0.0619 (8)	0.0314 (6)	−0.0014 (6)	0.0057 (5)	−0.0013 (5)
C12	0.0457 (7)	0.0524 (7)	0.0306 (6)	0.0017 (6)	0.0040 (5)	−0.0013 (5)
C1	0.0373 (6)	0.0405 (6)	0.0267 (5)	0.0026 (5)	0.0092 (4)	−0.0012 (4)
C13	0.0460 (7)	0.0582 (8)	0.0308 (6)	−0.0006 (6)	0.0058 (5)	−0.0010 (5)
C8	0.0366 (6)	0.0470 (7)	0.0302 (6)	0.0069 (5)	0.0044 (5)	0.0026 (5)
C2	0.0396 (6)	0.0404 (6)	0.0309 (5)	0.0014 (5)	0.0131 (5)	−0.0049 (5)
C10	0.0432 (6)	0.0482 (7)	0.0300 (6)	0.0046 (5)	0.0025 (5)	0.0010 (5)
C17	0.0441 (7)	0.0647 (9)	0.0326 (6)	−0.0039 (6)	0.0075 (5)	−0.0003 (6)
C14	0.0478 (7)	0.0579 (8)	0.0314 (6)	−0.0002 (6)	0.0034 (5)	−0.0018 (5)
C16	0.0473 (7)	0.0597 (8)	0.0330 (6)	−0.0001 (6)	0.0048 (5)	−0.0016 (6)
C6	0.0591 (8)	0.0471 (7)	0.0393 (6)	0.0016 (6)	0.0210 (6)	0.0022 (5)
C3	0.0392 (6)	0.0564 (8)	0.0443 (7)	−0.0002 (5)	0.0135 (5)	−0.0078 (6)
C5	0.0690 (9)	0.0500 (8)	0.0572 (8)	−0.0089 (7)	0.0382 (7)	−0.0030 (6)
C18	0.0494 (8)	0.0736 (10)	0.0394 (7)	0.0005 (7)	0.0021 (6)	0.0011 (7)
C19	0.0585 (9)	0.1111 (14)	0.0367 (7)	−0.0096 (9)	0.0006 (7)	0.0009 (8)
C4	0.0502 (8)	0.0569 (8)	0.0632 (9)	−0.0117 (6)	0.0300 (7)	−0.0127 (7)

### Geometric parameters (Å, º)

**Table:**

N2—C1	1.3759 (15)	C8—H8A	0.9700
N2—C7	1.3899 (15)	C8—H8B	0.9700
N2—C8	1.4550 (14)	C2—C3	1.3816 (17)
N1—C1	1.3688 (14)	C10—H10A	0.9700
N1—C2	1.3827 (15)	C10—H10B	0.9700
N1—H1	0.8600	C17—C18	1.5095 (18)
O1—C1	1.2314 (14)	C17—C16	1.5156 (18)
C11—C10	1.5184 (16)	C17—H17A	0.9700
C11—C12	1.5187 (16)	C17—H17B	0.9700
C11—H11A	0.9700	C14—H14A	0.9700
C11—H11B	0.9700	C14—H14B	0.9700
C7—C6	1.3771 (18)	C16—H16A	0.9700
C7—C2	1.3986 (16)	C16—H16B	0.9700
C9—C8	1.5177 (16)	C6—C5	1.3885 (19)
C9—C10	1.5218 (16)	C6—H6	0.9300
C9—H9A	0.9700	C3—C4	1.381 (2)
C9—H9B	0.9700	C3—H3	0.9300
C15—C16	1.5157 (18)	C5—C4	1.383 (2)
C15—C14	1.5195 (18)	C5—H5	0.9300
C15—H15A	0.9700	C18—C19	1.511 (2)
C15—H15B	0.9700	C18—H18A	0.9700
C12—C13	1.5180 (17)	C18—H18B	0.9700
C12—H12A	0.9700	C19—H19A	0.9600
C12—H12B	0.9700	C19—H19B	0.9600
C13—C14	1.5186 (17)	C19—H19C	0.9600
C13—H13A	0.9700	C4—H4	0.9300
C13—H13B	0.9700		
			
C1—N2—C7	109.94 (9)	N1—C2—C7	106.97 (10)
C1—N2—C8	123.45 (10)	C11—C10—C9	114.11 (11)
C7—N2—C8	126.15 (10)	C11—C10—H10A	108.7
C1—N1—C2	110.23 (9)	C9—C10—H10A	108.7
C1—N1—H1	124.9	C11—C10—H10B	108.7
C2—N1—H1	124.9	C9—C10—H10B	108.7
C10—C11—C12	113.25 (11)	H10A—C10—H10B	107.6
C10—C11—H11A	108.9	C18—C17—C16	114.41 (12)
C12—C11—H11A	108.9	C18—C17—H17A	108.7
C10—C11—H11B	108.9	C16—C17—H17A	108.7
C12—C11—H11B	108.9	C18—C17—H17B	108.7
H11A—C11—H11B	107.7	C16—C17—H17B	108.7
C6—C7—N2	131.94 (11)	H17A—C17—H17B	107.6
C6—C7—C2	121.55 (11)	C13—C14—C15	114.31 (12)
N2—C7—C2	106.51 (10)	C13—C14—H14A	108.7
C8—C9—C10	111.39 (10)	C15—C14—H14A	108.7
C8—C9—H9A	109.3	C13—C14—H14B	108.7
C10—C9—H9A	109.3	C15—C14—H14B	108.7
C8—C9—H9B	109.3	H14A—C14—H14B	107.6
C10—C9—H9B	109.3	C17—C16—C15	114.33 (12)
H9A—C9—H9B	108.0	C17—C16—H16A	108.7
C16—C15—C14	114.07 (12)	C15—C16—H16A	108.7
C16—C15—H15A	108.7	C17—C16—H16B	108.7
C14—C15—H15A	108.7	C15—C16—H16B	108.7
C16—C15—H15B	108.7	H16A—C16—H16B	107.6
C14—C15—H15B	108.7	C7—C6—C5	117.30 (12)
H15A—C15—H15B	107.6	C7—C6—H6	121.3
C13—C12—C11	114.18 (11)	C5—C6—H6	121.3
C13—C12—H12A	108.7	C4—C3—C2	117.68 (12)
C11—C12—H12A	108.7	C4—C3—H3	121.2
C13—C12—H12B	108.7	C2—C3—H3	121.2
C11—C12—H12B	108.7	C4—C5—C6	121.22 (13)
H12A—C12—H12B	107.6	C4—C5—H5	119.4
O1—C1—N1	127.98 (10)	C6—C5—H5	119.4
O1—C1—N2	125.67 (10)	C17—C18—C19	114.10 (14)
N1—C1—N2	106.35 (10)	C17—C18—H18A	108.7
C12—C13—C14	113.68 (12)	C19—C18—H18A	108.7
C12—C13—H13A	108.8	C17—C18—H18B	108.7
C14—C13—H13A	108.8	C19—C18—H18B	108.7
C12—C13—H13B	108.8	H18A—C18—H18B	107.6
C14—C13—H13B	108.8	C18—C19—H19A	109.5
H13A—C13—H13B	107.7	C18—C19—H19B	109.5
N2—C8—C9	113.73 (10)	H19A—C19—H19B	109.5
N2—C8—H8A	108.8	C18—C19—H19C	109.5
C9—C8—H8A	108.8	H19A—C19—H19C	109.5
N2—C8—H8B	108.8	H19B—C19—H19C	109.5
C9—C8—H8B	108.8	C3—C4—C5	121.52 (13)
H8A—C8—H8B	107.7	C3—C4—H4	119.2
C3—C2—N1	132.32 (11)	C5—C4—H4	119.2
C3—C2—C7	120.71 (12)		
			
C1—N2—C7—C6	−179.18 (12)	N2—C7—C2—C3	178.75 (10)
C8—N2—C7—C6	8.5 (2)	C6—C7—C2—N1	179.65 (10)
C1—N2—C7—C2	0.61 (13)	N2—C7—C2—N1	−0.17 (12)
C8—N2—C7—C2	−171.76 (10)	C12—C11—C10—C9	174.05 (11)
C10—C11—C12—C13	−176.14 (11)	C8—C9—C10—C11	176.53 (11)
C2—N1—C1—O1	−179.33 (11)	C12—C13—C14—C15	−179.52 (12)
C2—N1—C1—N2	0.70 (12)	C16—C15—C14—C13	−178.39 (12)
C7—N2—C1—O1	179.22 (11)	C18—C17—C16—C15	−178.41 (13)
C8—N2—C1—O1	−8.16 (18)	C14—C15—C16—C17	179.67 (12)
C7—N2—C1—N1	−0.81 (12)	N2—C7—C6—C5	−179.26 (12)
C8—N2—C1—N1	171.81 (10)	C2—C7—C6—C5	0.99 (18)
C11—C12—C13—C14	−179.29 (11)	N1—C2—C3—C4	179.30 (12)
C1—N2—C8—C9	104.30 (13)	C7—C2—C3—C4	0.70 (18)
C7—N2—C8—C9	−84.31 (15)	C7—C6—C5—C4	0.1 (2)
C10—C9—C8—N2	174.01 (10)	C16—C17—C18—C19	179.99 (13)
C1—N1—C2—C3	−179.08 (12)	C2—C3—C4—C5	0.4 (2)
C1—N1—C2—C7	−0.33 (13)	C6—C5—C4—C3	−0.9 (2)
C6—C7—C2—C3	−1.43 (18)		

### Hydrogen-bond geometry (Å, º)

**Table:**

*D*—H···*A*	*D*—H	H···*A*	*D*···*A*	*D*—H···*A*
N1—H1···O1^i^	0.86	1.97	2.815 (1)	168

Symmetry code: (i) −*x*, −*y*+2, −*z*.
